# Viral Quasispecies Inference from Single Observations—Mutagens as Accelerators of Quasispecies Evolution

**DOI:** 10.3390/microorganisms13092029

**Published:** 2025-08-30

**Authors:** Josep Gregori, Miquel Salicrú, Marta Ibáñez-Lligoña, Sergi Colomer-Castell, Carolina Campos, Alvaro González-Camuesco, Josep Quer

**Affiliations:** 1 Liver Diseases-Viral Hepatitis, Liver Unit, Vall d’Hebron Institute of Research (VHIR), Vall d’Hebron Barcelona Hospital Campus, Passeig Vall d’Hebron 119-129, 08035 Barcelona, Spain; marta.ibanez@vhir.org (M.I.-L.); sergi.colomer@vhir.org (S.C.-C.); carolina.campos@vhir.org (C.C.); alvaro.gonzalez@vhir.org (A.G.-C.); 2 Statistics Department, Biology Faculty, University of Barcelona, 08028 Barcelona, Spain; msalicru@ub.edu; 3 Centro de Investigación Biomédica en Red de Enfermedades Hepáticas y Digestivas (CIBEREHD), Instituto de Salud Carlos III, Av. Monforte de Lemos, 3-5, 28029 Madrid, Spain; 4 Medicine Department, Universitat Autonoma de Barcelona (UAB), Campus de la UAB, Plaça Cívica, 08193 Bellaterra, Spain; 5 Biochemistry and Molecular Biology Department, Universitat Autonoma de Barcelona (UAB), Campus de la UAB, Plaça Cívica, 08193 Bellaterra, Spain

**Keywords:** inference, quasispecies, single-observation, RNA-virus, mutagens, inhibitors

## Abstract

RNA virus populations exist as quasispecies-complex, dynamic clouds of closely related but genetically diverse variants generated by high mutation rates during replication. Assessing quasispecies structure and diversity is crucial for understanding viral evolution, adaptation, and response to antiviral treatments. However, comparing single quasispecies observations from individual biosamples, especially at different infection or treatment time points, presents statistical challenges. Traditional inferential tests are inapplicable due to the lack of replicate observations, and resampling-based approaches such as the bootstrap and jackknife are limited by biases and non-independence, particularly for diversity indices sensitive to rare haplotypes. In this study, we address these limitations by applying the delta method to derive analytical variances for a set of quasispecies structure indicators specifically designed to assess the quasispecies maturation state. We demonstrate the utility of this approach using high-depth next-generation sequencing data from hepatitis C virus (HCV) quasispecies evolving in vitro under various conditions, including free evolution and exposure to antiviral or mutagenic treatments. Our results reveal that with highly fit HCV quasispecies, sofosbuvir inhibits quasispecies genetic diversity, while mutagenic treatments accelerate maturation, compared to untreated controls. We emphasize the interpretation of results through absolute differences, log-fold changes, and standardized effect sizes, moving beyond mere statistical significance. This framework enables robust, quantitative comparisons of quasispecies diversity from single observations, providing valuable insights into viral adaptation and treatment response. The R code and session info with required libraries and versions is provided in the supplementary material.

## 1. Introduction

A quasispecies is the complex and dynamic population of closely related but genetically diverse viral variants that arises during RNA virus infection. This population structure arises due to the high mutation rates of RNA viruses, which can introduce multiple errors during genome replication. The quasispecies consists of a mutant spectrum or cloud of viral genomes that are continuously generated and subject to selection within the host [1,2,3]. Quasispecies maturation is understood as an enrichment in genetic diversity caused by replication errors. This process facilitates the selection and proliferation of alternative genetic variants expressing functional phenotypes, resulting in enhanced quasispecies fitness, increased resilience and reduced susceptibility to antiviral treatments [4,5,6,7].

A quasispecies observation consists in sequencing to high depth a quasispecies sample taken from a biosample infected with a RNA virus, and it is represented by a set of genomes (haplotypes) and observed frequencies, in the form of NGS sequencing read counts. This observation constitutes a sample of size *n*, with *n* the number of final reads.

In the study of quasispecies structure and diversity, we are often confronted with a situation where we need to compare single quasispecies observations from single biosamples, possibly taken at two different infection or treatment time points. This situation presents statistic challenges, because traditional inferencial tests are not applicable when comparing diversity indices or structure indicators of single quasispecies observations. A useful test must be based on estimates of expected diversity and its variability for each of the quasispecies to be compared. Analytical approximations based on the Delta metod are available for any index or indicator expressed as a function of multinomial proportions [8,9,10]. Besides, we know that some diversity indices, which may be of interest, are sensitive to sample size differences, which means that unbalanced samples will require normalization. We focus on diversity indices which may be calculated directly from the vector of haplotype NGS read counts.

Alternative methods rely on tools based on resampling to generate empirical distributions from which estimate standard errors, confidence intervals, and *p*-values. A resampling method commonly used to generate such distributions to estimate variances is the bootstrap [11,12,13], where at each resampling cycle, we sample with replacement a number of reads equal to the sample size. At each bootstrap cycle diversity indices are computed, and at the end the estimated values and corresponding variances result from the mean and variance of the observed values in the B bootstrap cycles. However we know that the bootstrap suffers from the 0.632 limitation, which implies that in each resampling cycle with replacement at the full sample size, at most 63.2% of the reads in the sample are observed, meaning that 36.8% of the reads are replicates of already observed reads, and likewise that 36.8% of the reads in the quasispecies sample remain unobserved [14]. This limitation results in biases in the estimation of diversity indices sensitive to low and very low haplotype frequencies. Particularly affected are the fraction of singletons and rare haplotypes, the total number of haplotypes and indices such as Shannon entropy or the Hill number at q = 1 [14]. Note that any approach based on parametric multinomial resampling is submitted to the same limitations; resampling from a multinomial given its parameters and a sample size, is equivalent to resampling with replacement.

An alternative to the bootstrap is the jackknife [11]. While similar to the bootstrap in that it involves resampling, the key difference is that the jackknife samples without replacement, which aligns with our requirements. However, it is well known that the traditional delete-1 jackknife estimator performs poorly with non-smooth estimators, those sensitive to one or a few values in the dataset, such as the median or other empirical quantiles. This makes it unsuitable for computing confidence intervals. It has been shown that the delete-d Jackknife [11], where d/n→δ∈(0,1) asymptotically (rather than the overly restrictive $\sqrt{n}<d<\left(n-1\right)$), is a consistent estimator [11,15] and overcomes most of the limitations in the delete-1 approach. This method resembles the delete-1 estimator but incorporates a normalization constant accounting for the number of distinct subsets with *d* items removed nd, However, computational complexity increases rapidly with larger *d*, especially for large n. To address this, the estimator is often evaluated using a subset of all possible nd sets [11,16,17]. A key limitation is that estimated variances depend on *d*, the number of reads removed in each iteration. Furthermore, the jackknife values generated under this scheme are not independent and identically distributed (i.i.d.), as resampled subsets overlap. Having i.i.d. values is a fundamental requirement for obtaining *p*-values or confidence intervals from empirically distributed values.

We find ourselves restricted to the use of the delta method, as no resampling scheme appears to be free of significant limitations in our context.

We present under Methods a set of quasispecies structure indicators specifically devised to assess the maturation state of a quasispecies [7] along with their analytical variances obtained using the delta method [18,19,20]. The Results section illustrates the application of the proposed method to test data of HCV quasispecies evolving in vitro in a non-coevolving environment, either freely or under antiviral treatments [21,22,23,24]. Treatments are administered to high-fitness quasispecies evolved over 100 passages in a non-coevolving environment, which show reduced response to antiviral treatments. These illustrative results demonstrate sofosbuvir’s inhibitory effects on quasispecies genetic structure and the accelerated quasispecies maturation caused by mutagen treatments in monotherapy, compared to treatment-free evolution in a non-coevolving environment. We focus on interpreting the results by integrating absolute differences, log-fold changes, and standardized effect sizes, moving beyond statistical significance alone—which is almost guaranteed by the large sample sizes. It must be emphasized, however, that the cell-culture data presented here serve only as an example of the method’s applicability. While the observed trends are consistent with findings from clinical datasets [4,5,6,7], these data alone do not support sufficiently robust conclusions, and further studies—including biological replicates of cell-culture experiments—will be needed. The directions of the effects are clear, but their magnitudes may vary across independent studies.

## 2. Materials and Methods

### 2.1. Quasispecies Maturity Indicators

Quasispecies maturation reflects a global trend of within-host quasispecies evolution toward enriched genetic diversity and phenotypic redundancy, driven by continuous emergence and selection of new functional variants with fitness advantages [7]. Table 1 shows the set of selected quasispecies structure indicators to account for the evolutionary state of a quasispecies in its path from a founding quasispecies, A, dominated by a highly abundant master genome and a peaked fitness landscape, towards its hypothetical end-point, Z, in the form of a flattened fitness landscape, with a high number of genomes at low and equivalent abundances, in dynamic equilibrum, with no dominance [5,6,7,25].

The zeroes for TopN and Master, in State Z, should be interpreted as an infinitesimal frequency ϵ, that is ϵ>0 and ϵ<1/n for a big positive integer *n*, rather than strictly zero. In biochemical terms this ϵ may be interpreted as one to a few or several molecules, in the context of a high viral titer. Similarly, the zeroes for Rare1 and Rare2, in state A, should be interpreted as a result of several variants other than the master haplotype, at infinitesimal frequencies, ϵ. However, we represent ϵ formally as 0.

A global quasispecies maturity score for a given quasispecies, *Qs*, may be computed as the distance of this quasispecies from State A, $d_{A}$, in the multidimensional space represented by this set of indicators.

(1) $$d_{A}\left(Qs\right)=\sqrt{\sum_{i}\left[{\left(f_{i}\left(Qs\right)-f_{i}\left(A\right)\right)}^{2}\right]}$$

where $f_{i}$ is the set of maturity indicators. This score may be normalized to the range 0–1 as $d_{N}\left(Qs\right)=d_{A}\left(Qs\right)/d_{A}\left(Z\right)$. Whereas each indicator refers to a given aspect of quasispecies structure, $d_{N}$ represents the combined effect of all changes in pushing the quasispecies towards its purest origin, A, or its fate, Z [7].

### 2.2. Delta Method

The delta method [18,19] provides a statistical foundation for justifying normality in tests involving functions of random variables by leveraging the asymptotic normality of the parameters estimators. When an estimator is asymptotically normal, the delta method allows us to approximate the distribution of smooth (i.e. differentiable) functions of that estimator as also normal. The proposed quasispecies structure indicators are based on the distribution of haplotypes, which follows a multinomial distribution with as many categories and parameters as there are haplotypes. Multinomial proportions estimated from a sample exhibit asymptotically normal behavior,

$$\sqrt{n}\left(\hat{P}-P\right)\overset{d}{\to }N\left(0,\Sigma_{P}\right),\;\;\Sigma_{P}=\left(\sigma_{ij}\left(P\right)\right)=\left(\delta_{ij}p_{i}-p_{i}p_{j}\right)$$

providing the basis for applying the delta method to functions of estimated proportions $\hat{P}:\left\{{\hat{p}}_{i};\;i=1\ldots H\right\}$, and proportion aggregates, and for conducting tests based on the normal distribution using these indicators. In what follows, let $\hat{P}$ denote the vector of estimated haplotype frequencies, sorted in decreasing order of frequency without loss of generality, so that $\hat{p_{1}}$ represents the estimated frequency of the master haplotype.

Breavly, if an estimator $\hat{\Theta }=\left({\hat{\theta }}_{1},{\hat{\theta }}_{2},\ldots ,{\hat{\theta }}_{H}\right)$ satisfies

$$\sqrt{n}\left(\hat{\Theta }-\Theta \right)\overset{d}{\to }N\left(0,\Sigma_{\Theta }\right),\;\;\Sigma_{\Theta }=\left(\sigma_{ij}\left(\Theta \right)\right)$$

then for a differentiable function *g*, the delta method ensures:

$$\sqrt{n}\left(g\left(\hat{\Theta }\right)-g\left(\Theta \right)\right)\overset{d}{\to }N\left(0,g^{\prime }{\left(\Theta \right)}^{T}\;\Sigma_{\Theta }\;g^{\prime }\left(\Theta \right)\right)$$

provided that $g^{\prime }{\left(\Theta \right)}^{T}\;\Sigma_{\Theta }\;g^{\prime }\left(\Theta \right)>0$, being

$$g^{\prime }{\left(\Theta \right)}^{T}=\left(\frac{\partial g(\Theta )}{\partial \theta_{1}},\ldots ,\frac{\partial g(\Theta )}{\partial \theta_{H}}\right)$$

This allows normality tests to be applied to $g(\hat{\Theta })$, even if *g* is non linear.

### 2.3. Indicators’ Variance and Standard Error by the Delta Method

The method approximates the standard error of $g(\hat{P})$ of first order Taylor expansion:

$$\begin{array}{c} \mathrm{SE}{\left(g\left(\hat{P}\right)\right)}^{2}=\frac{1}{n}\mathrm{Var}\left(g\left(\hat{P}\right)\right)\approx \frac{1}{n}g^{\prime }{\left(P\right)}^{T}\;\Sigma_{P}\;g^{\prime }\left(P\right)\approx \\ \approx \frac{1}{n}g^{\prime }{\left(\hat{P}\right)}^{T}\;\Sigma_{\hat{P}}\;g^{\prime }\left(\hat{P}\right) \end{array}$$

where $\hat{P}$ is an estimated binomial or multinomial proportion, a vector of multinomial proportions, or an aggregate of them.

The full set of analytically derived equation of the variances for all proposed quasispecies structure indicators using the delta method is provided in the Supplementary Material [18,19,20,26].

### 2.4. Large Sample Sizes and p-Values

With sample sizes on the order of 100,000 reads, even minuscule differences in diversity measures between two quasispecies can achieve statistical significance due to the extremely small standard errors. However, statistical significance alone does not guarantee biological or practical relevance. In such cases, 99% confidence intervals help quantify the precision of diversity estimates and delineate the range of plausible values for the true difference between quasispecies. Nevertheless, the primary emphasis should be placed on the magnitude of the observed effect rather than solely on statistical significance. While estimated absolute differences, relative differences, or ratios between indicators of the quasispecies provide useful metrics, these values are not always readily interpretable in biological or clinical contexts. This highlights the need for standardized effect sizes, which allow for meaningful comparison across studies and contexts by expressing the magnitude of differences in a scale-independent manner. Standardized effect sizes facilitate the assessment of whether observed differences are not only statistically significant, but also biologically or clinically meaningful, thereby enhancing the interpretability and generalizability of findings. Effect size measures, like Cohen’s d, can provide more meaningful information about the practical importance of observed differences, and are independent of sample sizes [27,28,29,30]. While they may not substitute for biological significance when sufficient knowledge is available, they do offer a generally interpretable scale of magnitudes [27,30].

### 2.5. Effect Size

Effect sizes quantify the magnitude of effects (i.e., strength of a relationship, size of a difference), which are the outcomes of our empirical research. These measures are independent of sample sizes, contrary to *p*-values.

In general terms, given a quasispecies indicator *H* and the standard error of its estimator, ${\mathrm{SE}}^{2}$, the effect size for the difference of two quasispecies $H_{A}-H_{B}$, corresponds to:

(2) $$\begin{array}{c} d_{H}=\frac{H_{A}-H_{B}}{\sqrt{(n_{A}\cdot {\mathrm{SE}}_{A}^{2}+n_{B}\cdot {\mathrm{SE}}_{B}^{2})/2}}=\\ =\frac{H_{A}-H_{B}}{\sqrt{(\sigma_{A}^{2}+\sigma_{B}^{2})/2}}=\frac{H_{A}-H_{B}}{S_{p}} \end{array}$$

It follows from the expression relating the standard error of an estimator and its original standard deviation $\sigma^{2}/n={\mathrm{SE}}^{2}$.

Cohen’s *d* is a unitless, standardized measure of effect size that quantifies the difference between value estimates in two groups in terms of standard deviation units. When the data is normally distributed, Cohen’s *d* can be interpreted in probabilistic terms, which offer a more intuitive interpretation. Two probabilistic metrics can be used: the non-overlap (NOV) of the two distributions and the probability of superiority of one condition over the other. Guidelines for interpreting *d* values using common terms of magnitude, as well as their probabilistic equivalents are given in Supplementary Table S1 [30].

### 2.6. Z, t, and Cohen’s d

With unequal variances the comparison of two estimates is made using the Welch *t*-test, where the *t*-statistic is defined as:

(3) $$t=\frac{{\hat{H}}_{A}-{\hat{H}}_{B}}{\sqrt{{\mathrm{SE}}_{A}^{2}+{\mathrm{SE}}_{B}^{2}}}=\frac{{\hat{H}}_{A}-{\hat{H}}_{B}}{\sqrt{s_{A}^{2}/n+s_{B}^{2}/n}}=d\;\sqrt{\frac{n}{2}}$$

where *d* is the standardized difference based on pooled variance, with $n_{a}=n_{b}=n$. With a reference size of 100,000 reads, the above equation becomes:

t=224×d

When the indicator *H* corresponds to a proportion, or to an aggregate of frequencies from a multinomial distribution, $\hat{p}$, with a reference sample size of n=100,000 reads, we can use the normal approximation to a proportion:

$$Z=224\times \frac{{\hat{p}}_{A}-{\hat{p}}_{B}}{\sqrt{\bar{p}\left(1-\bar{p}\right)}}=245\times d$$

where $\bar{p}=\frac{1}{2}\left({\hat{p}}_{A}+{\hat{p}}_{B}\right)$, when $n_{a}=n_{b}=n$, and *d* is the standardized difference based on the pooled variance. The biggest denominator in this equation occurs for $\bar{p}=0.5$, giving 0.5, then:

$$Z\geq 448\times ({\hat{p}}_{A}-{\hat{p}}_{B})$$

The critical *Z* value for α=0.0005 is 3.29. Therefore, a difference in proportion greather than 3.29/448=0.0073 will be statistically significant at 99.9% level, regardless of the $\bar{p}$ value.

Note, however, that the probability density function (PDF) of the t-distribution simplifies to the standard normal PDF as the degrees of freedom tend to infinity. For very high sample sizes, *n*, the t-distribution for the difference of means converges to the standard normal distribution due to the Central Limit Theorem and the reduced uncertainty in estimating the population standard deviation. Therefore, a z-test is acceptable for all proposed quasispecies indicators in such cases.

### 2.7. Quasispeces Samples, Cells, Viruses, and Drugs

This is a reanalysis of data in previously published works [21,22] that investigates the evolution of HCV viral quasispecies under various conditions in cell culture, including natural passage, direct-acting antiviral (DAA) treatment, and mutagenic agents.

HCV RNA expressed from plasmid Jc1FLAG2(p7-nsGluc2A) (genotype 2a) [31] was transfected into Huh-7 Lunet cells and amplified in Huh-7.5 cells to produce the initial virus population HCV p0 [32]. HCV p0 was subjected to 100 serial passages in Huh-7.5 reporter cells in the absence of any drug, equivalent to ≈300 days of evolution. The populations at passage 100 (HCV p100) displayed increased replication in Huh-7.5 reported cells [23,33], with increased fitness 2.2-fold relative to HCV p0 [22,33]. The 100 passages are equivalent to about a year of continuous evolution in a non-coevolving environment [23,24] free of external pressures.

HCV p100 was further passaged in the absence of any drug (Ctl), in the presence of favipiravir (FPV, 400 μM), ribavirin (RBN, 100 μM), and sofosbivir (SOF, 800 nM), for extra ten passages (30 days). The drug concentrations were chosen for their capacity to extinguish HCV p0 in four to five passages, under the infection conditions used in the experiments [22]. Favipiravir (T-705) (Atomax Chemicals Co., Ltd., Hong Kong, China), ribavirin (Sigma, Hong Kong, China), and sofosbuvir (Selleck Chemicals, Houston, TX, USA) were prepared and used as previously described [21,34,35].

To achieve a comprehensive picture of quasispecies composition, our previous results have underlined the need to perform deep-sequencing studies with very high coverage (over $1\times 10^{5}$ reads per amplicon) and avoid unnecessary abundance-based filter [5,6,7,25,36]. This is particularly important with mutagenic treatments, where their effect in the short term is mainly observed at the lowest level of haplotype frequencies [5,36].

A NS5B amplicon spanning positions 8552:8869 is deeply sequenced. Sequencing was performed using MiSeq Illumina^®^ instruments, in 2 × 300 paired-end mode, obtaining deep coverages ranging from 110 K to 191 K per strand, amplicon and sample (Table 2).

### 2.8. Implementation

The described statistic methods have been implemented in R functions. R code and session info with R and libraries versions are provided in the Supplementary Material. Given a quasispecies represented by a vector of haplotype frequencies as read counts or relative frequencies, these functions calculate quasispecies indicator values and corresponding variances. These are then used to compare and test pairs of quasispecies. The test provides for each indicator the following information: observed difference, observed ratio, z-statistic, Benjamini-Hochberg multi-test adjusted *p*-values, Conhen’s d, NOV and PS.

## 3. Results

### 3.1. Maturity Indicator Values on Rarefied Quasispecies

All quasispecies samples are normalized by rarefaction [14] to a reference size of 110,000 reads -the minimum size to include all samples- the median values after 500 cycles of rarefaction are taken for each indicator (Table 3).

The calculated variances from the analytical expressions are displayed in Supplementary Table S2.

**Figure 1:**
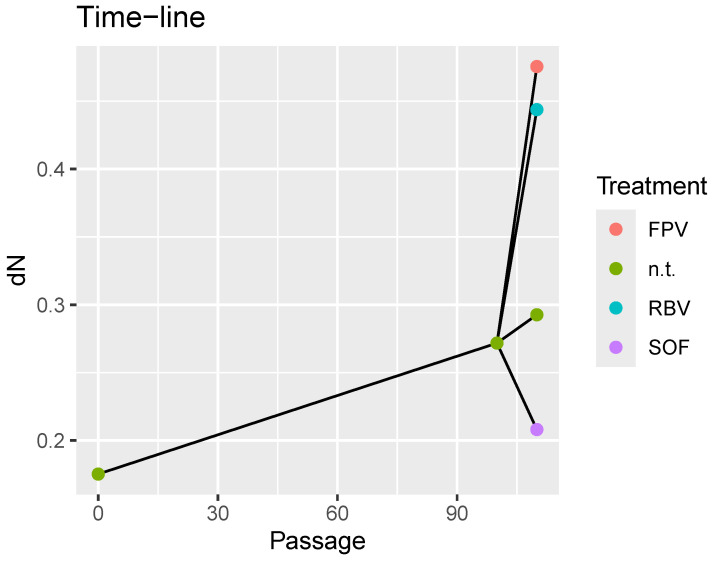
Treatments time-line, showing the effects of each treatment on the maturity score dN over time.

Figure 1 provides a summary of the treatment effects on the maturity score dN, displaying its evolution along the cell-culture timeline. From p100, ten extra passages without treatment are used as control, to be compared with the results of ten passages, starting at p100, treated with SOF, RBV or FPV in monotherapy. Clearly the treatments with mutagens in ten passages from p100, resulted in accelerated changes on dN, greater than those achieved in the 100 passages from p0 of natural evolution in cell culture.

### 3.2. Tests

The changes in quasispecies structure indicators and in quasispeces maturity score are evaluated and tested between the following states:

- Pass 0 (p0) quasspecies versus pass 100 (p100), with no treatment in between (Supplementary Table S3).
- Pass 110 (p110, Ctl), with extra 10 passages to p100, versus p100, with p100 used as treatment baseline (Supplementary Table S4)
- Ten passages of treatment with sofosbuvir (SOF), starting at p100, versus the base line p100 (Supplementary Table S5).
- Ten passages of treatment with ribavrine (RBV), starting at p100, versus the base line p100 (Supplementary Table S6).
- Ten passages of treatment with favipiravir (FPV), starting at p100, versus the base line p100 (Supplementary Table S7).

From these tests we obtain *p*-values and effect sizes of different treatments over ten passages, starting at p100, on quasispecies structure indicators. Note that virtually all adjusted *p*-values across all comparisons are extremely low due to the very large sample sizes involved. The lowest *t* statistic (in absolute value) among all tests performed is 5.79 (see Supplementary Tables S3–S7), making the p-value in this context almost meaningless. Correspondingly, the 99.9% confidence intervals are very narrow, as illustrated in Supplementary Figure S4 for $d_{N}$.

**Figure 2:**
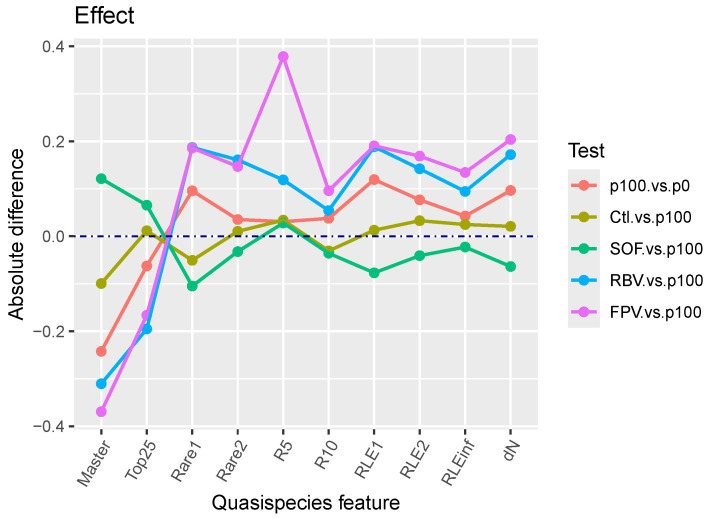
Observed differences in quasispecies indicators between biological conditions.

**Figure 3:**
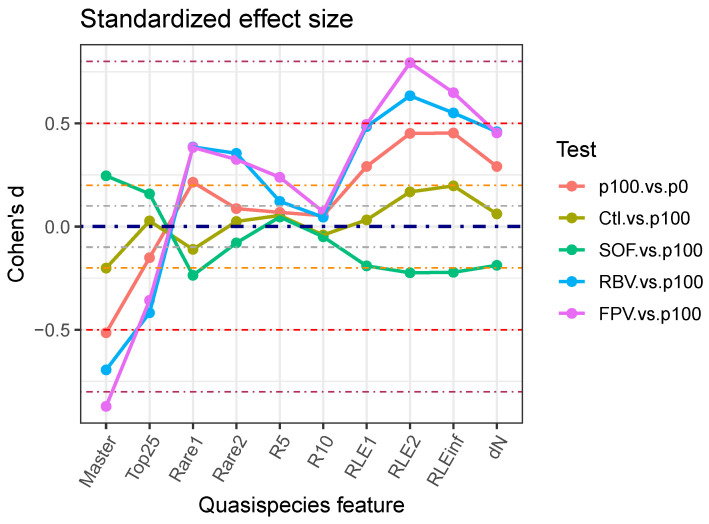
Cohens’ d standardized effect size of treatments. Dash-dot lines: effect magnitude borders for tiny 0.0–0.1, very small 0.1–0.2, small 0.2–0.5, and moderate 0.5–0.8.

Effect sizes provide better alternatives for interpreting test results. Figure 2 shows the observed differences between quasispecies pairs, whereas Figure 3 and Supplementary Figure S1 display the resulting Cohen’s *d* values for each treatment, indicating effect magnitudes. Supplementary Figures S2 and S3 present these effects as PS and NOV values, respectively.

Let us take as reference the changes observed in 100 passages, from HCV p0 to HCV p100. All differences of indicators are highly statistically significant, with absolute *t* values above 12.3, and practically null *p*-values (Supplementary Table S3).

The effect size values for $d_{N}$ gives the magnitude of the effect of treatment on all combined quasispecies indicators. Supplementary Table S8 displays the test results on *dN*, where all adjusted *p*-values are 0. Supplementary Figure S5 depicts these values for each treatment in a barplot, with the ratio to the p100.vs.p0 *d* value shown inside the bars, where the magnitude of each treatment effect is referred to that of the natural evolution from HCV p0 to HCV p100.

Sofosbuvir is an inhibitor, a direct acting antiviral (DAA), Ribavirine and Favipiravir act as mutagens, increasing the virus replication error rate. The mutagens acted as accelerators of quasispecies evolution without causing error catastrophe or extinction, as viral titer was maintained and even slightly increased throughout the treatments. The genetic diversification, compatible with viral functionality, achieved in ten passages with mutagenic treatment was higher than that observed in 100 passages from HCV p0 to HCV p100, with an effect size on the maturity score dN over 1.5 times that observed in the natural evolution from HCV p0 to HCV p100 (Supplementary Figure S5). Although the Ribavirine and Favipiravir dosage was sufficient to drive HCV p0 to extinction within five passages as previously published in [22], the increased fitness achieved by HCV p100 [22,33] was enough to counteract replication inhibition. The sustained viral titer, combined with the elevated error rate, enabled the production and selection of multiple variants with comparable fitness, resulting in a more mature quasispecies.

The treatment with sofosbuvir resulted in a contraction of the genetic diversity, with increased top haplotypes dominance, and reduced fraction of reads for haplotypes below 1% frequency. The contraction in dN was moderate. Note that all other treatments resulted in increases of dN (Supplementary Figure S5, and Supplementary Table S8).

## 4. Discussion

Quasispecies fitness has been shown as a determinant of lower sensitivity and failure to antiviral treatments, beyond the presence of resistance mutations, both in HCV cell culture experiments [21,22,23,24] and in HCV chronically infected patients [7]. Furthermore ribavirine has been shown to induce highly resistant quasispecies in immunocompromised patients chronically infected with HEV, with no response to increased doses [5,6]. We developed a statistical framework based on the Delta method and the asymptotic normality of multinomial parameters, and implemented it with R code, allowing to assess changes in quasispecies genetic structure indicators from single quasispecies observations sequenced at high depth. The quasispecies maturity state, understood as a surrogate of quasispecies fitness, may be taken as a general biomarker of resilience to antiviral treatments.

This study provides a robust analytical framework for assessing the structure and maturation of RNA virus quasispecies from single high-depth sequencing observations, overcoming the limitations of traditional resampling-based statistical methods in this field. By leveraging the delta method for analytical variance estimation, we enable meaningful comparisons of quasispecies diversity and structure in scenarios where replicate samples are unavailable, a common situation in virological studies. Like in -omic experiments, to mitigate the impact of sources of variability external to sampling itself—such as batch effects, confounding factors, and technical variability in general—careful experimental design is essential [37,38,39]. This includes balanced blocking, randomization, and parallel processing within a short time window whenever possible. Ideally, all quasispecies to be compared should be sequenced in the same instrument run, or in balanced runs under the conditions being compared.

Our results offer clear empirical support for the anticipated effects of different evolutionary pressures on quasispecies structure. As expected, prolonged evolution in a non-coevolving environment (100 passages, HCV p0 to p100) led to increased genetic diversity, reduced dominance of the master haplotype, and greater evenness among haplotype frequencies. These changes are consistent with the theoretical understanding that high mutation rates and relaxed selective constraints facilitate the exploration of sequence space, promoting the emergence and coexistence of multiple functional variants. This genomic plasticity enhances the viral capacity for persistence and transmission, by enabling the virus to evade selective pressures, particularly immune responses, antiviral treatments, and other host-related constraints. This strategy not only complicates eradication efforts but may also promote the evolution of escape mutants, ultimately undermining therapeutic efficacy and long-term immune control. The observed increase in fitness and quasispecies maturity aligns with previous findings, emphasizing the adaptability and evolutionary potential of RNA viruses under stable conditions.

In contrast, treatment with the polymerase inhibitor sofosbuvir (SOF) produced a marked contraction in genetic diversity, as reflected by the inverse changes in all diversity and evenness indicators. This reduction in diversity, despite stable viral titers, suggests that SOF imposes strong selective pressure, favoring the survival of a subset of fit variants with low susceptibility and response to the treatment. The maintenance of viral load under these conditions highlights the capacity of highly adapted quasispecies to withstand antiviral intervention, likely through the rapid selection of pre-existing or emergent resistant haplotypes.

Mutagenic treatments with ribavirin (RBV) and favipiravir (FPV) had the opposite effect, accelerating quasispecies maturation in 10 passages beyond that observed during 100 passages of natural evolution. Both mutagens significantly increased diversity and evenness, with FPV exerting the strongest effect. Notably, these treatments did not lead to error catastrophe or viral extinction in the high-fitness HCV p100 background, underscoring the resilience of mature quasispecies populations. The ability of these populations to maintain or even increase viral titers under elevated mutational pressure suggests that high fitness confers a buffer against mutagen-induced deleterious effects, enabling the continued generation and selection of fit variants.

The goal of treating a viral infection with mutagenic agents is to achieve a cure through lethal mutagenesis [40]. During lethal mutagenesis, there is typically an initial increase in genetic diversity, followed by a sharp decline in viral titer as the viral population approaches extinction. This occurs because the mutation rate surpasses the error threshold, leading to a loss of genetic information and viability. Consequently, deleterious mutations accumulate, ultimately causing population collapse [40,41,42]. One of the HCV in vitro studies from which we have reanalyzed data reported that high-fitness hepatitis C virus, such as HCV p100, was resistant to lethal mutagenesis [22]. Furthermore, in two recent studies [5,6], we observed that repeated unsuccessful treatments with ribavirin in two chronically infected, immunosuppressed HEV patients resulted in highly mature quasispecies that were resilient to the treatment. This suggests that under mutagenic treatment, specially in monotherapy, quasispecies may evolve in one of two opposing directions [4], depending on their global fitness, genetic and phenotypic distribution structure, viral load or population size, and treatment regimen: 1—towards lethal mutagenesis and extinction, or 2—towards enhanced quasispecies maturity with higher fitness and reduced susceptibility to further treatments [4,6,25].

It should be noted that the cell-culture data presented here are intended solely as an illustration of the method’s applicability. Although the observed trends are in agreement with results reported in clinical datasets, these findings alone are not sufficient to draw definitive conclusions. Additional investigations, including biological replicates of cell-culture experiments, will be required to strengthen the evidence. The overall patterns appear consistent, but the magnitude of the effects may differ across independent studies.

## 5. Conclusions

From a methodological perspective, our approach addresses a critical gap in the analysis of viral quasispecies, where single-sample comparisons are often the only option. However, it is important to note that these samples, despite being single observations, represent a quasispecies with a very high sample size, *n*, as the number of sequenced reads. The analytical variance estimates derived via the delta method offer a reliable basis for hypothesis testing and confidence interval construction, free from the biases and limitations inherent in bootstrap and jackknife resampling for quasisecies diversity indices.

In summary, this work advances the quantitative analysis of viral quasispecies by introducing a statistically rigorous and biologically insightful framework for single-sample comparisons. Our findings underscore the dynamic interplay between mutation, selection, and treatment in shaping viral populations, with important implications for understanding viral evolution, adaptation, and the emergence of resistance to antiviral therapies. Future research should aim to validate and extend these observations, as well as investigate the framework’s potential utility in clinical settings, where monitoring quasispecies diversity may help guide treatment strategies and predict therapeutic outcomes.

## Figures and Tables

**Table 1:** Selected maturity indicators. Description and expected values in the two limiting states [25].

Indicator	Description	State A	State Z
TopN	Fraction of reads for top N hpl.	1.00	0.00
Master	Dominant haplotype frequency	1.00	0.00
Rare1	Fraction of reads for hpl ≤1%	0.00	1.00
Rare2	Fraction of reads for hpl ≤0.1%	0.00	1.00
$RLE_{1}$	Relative logarithmic evennes at q=1	0.00	1.00
$RLE_{2}$	Relative logarithmic evennes at q=2	0.00	1.00
$RLE_{\infty }$	Relative logarithmic evennes at q=∞	0.00	1.00
$R_{k}$	Evenness among top k haplotypes	0.00	1.00

**Table 2:** Reads per sample. Forward strand only.

ID	Reads
p0	123,587
p100	179,382
Ctl	136,432
FPV	191,058
RBV	134,718
SOF	111,299

**Table 3:** (**A**)—Quasispecies maturity indicators. Rarefied values to 110,000 reads. (**B**)—Quasispecies maturity indicators, continued. Rarefied values to 110,000 reads.

(A)						
ID	nHpl	Master	Top25	Rare1	Rare2	dN
p0	4879.0	0.73728	0.80969	0.23231	0.19061	0.17525
p100	7883.5	0.49492	0.74722	0.32811	0.22594	0.27175
Ctl	8042.0	0.39545	0.75894	0.27743	0.23635	0.29263
SOF	5939.0	0.61619	0.81265	0.22331	0.19370	0.20805
RBV	17,702.5	0.18436	0.55210	0.51516	0.38691	0.44376
FPV	17,138.0	0.12553	0.58100	0.51404	0.37284	0.47553
(**B**)						
**ID**	**R5**	**R10**	**RLE1**	**RLE2**	**RLEinf**	**dN**
p0	0.01934	0.02206	0.27130	0.07163	0.03589	0.17525
p100	0.05016	0.05977	0.39069	0.14845	0.07840	0.27175
Ctl	0.08407	0.02905	0.40351	0.18128	0.10317	0.29263
SOF	0.07808	0.02405	0.31369	0.10767	0.05572	0.20805
RBV	0.16893	0.11401	0.57920	0.29061	0.17286	0.44376
FPV	0.42867	0.15571	0.58111	0.31728	0.21286	0.47553

## Data Availability

Sequence data that support the findings of this study was published in [22] and are openly available in the GenBank Sequence Read Archive database with BioProject “*Resistance of high fitness hepatitis C virus to lethal mutagenesis*” and accession PRJNA720288. The FASTA files with haplotypes and frequencies of each sample used in the study are given in a zipped file as Supplementary Material.

## Supplementary Materials

The following supporting information can be downloaded at: https://www.mdpi.com/article/10.3390/microorganisms13092029/s1, **Supplementary File S1**. **Supplementary methods and results**. A supplementary document with methods, equations, results, tables, and figures, including the R code, required libraries and versions. Follows a list with captions of tables and figures in this Supplementary File. **Table S1**. **Cohen’s *d* interpretation guidelines**. **Table S2**. **Variance of rarefied maturity indicators**. **Table S3**. **Test results**. p100 quasispecies versus p0. Dif: p100-p0, Ratio: p100/p0, adj.pval: BH adjusted *p*-values, d: Effect size, NOV: Non-overlap, PS: Probability of superiority. **Table S4**. **Test results**. p110 (Ctl) quasispecies versus p100. Dif: p110-100, Ratio: p110/p100. **Table S5**. **Test results**. Effect of 10 passes treated with Sofosbuvir, starting at p100. Dif: SOF-p100, Ratio: SOF/p100. **Table S6**. **Test results**. Effect of 10 passes treated with Ribavirine, starting at p100. Dif: RBV-p100, Ratio: RBV/p100. **Table S7**. **Test results**. Effect of 10 passes treated with Favipiravir, starting at p100. Dif: FPV-p100, Ratio: FPV/100. **Table S8**. **Summary of treatment results** on $d_{N}$, with dN.d.R the ratio of $d_{N}$*d* values to the p100.vs.p0 $d_{N}$*d* value. **Figure S1**. **Standardized effect size**. Cohens’ d standardized effect size of treatments. Backgound color depicts effect magnitude regions. Gray: tiny effect, orange: very small effect, pink: small effect, purple: moderate effect, white: large effect. **Figure S2**. **Probability of superiority**. Standardized effect size of treatments, evaluated in probabilitistic terms as probability of superiority. **Figure S3**. **Distributions non-overlap**. Standardized effect size of treatments, evaluated in probabilitistic terms as distributions non-overlap. **Figure S4**. **Distance from state A**. Maturity score as the normalized distance of the quasispecies from state A. With 99.9% CIs. Dash-dot line at p100 $d_{N}$ as reference. **Figure S5**. **Effect size on $d_{N}$ in each treatment**. Numbers inside bars: dN.d.R, ratio of $d_{N}$ *d* values to p100.vs.p0 *d* value. **Supplementary File S2**. **Fasta files zipped folder**. Zipped folder with the fasta files of all quasispecies in the study. Each sequence is a quasispecies haplotype, with headers expressing IDs, and frequencies as read counts and percentage. Refs. [6,30,43] are cited within.
