# Supplementary material for: Viral Quasispecies Inference from Single Observations—Mutagens as Accelerators of Quasispecies Evolution
Source: Microorganisms. 2025 Aug 30;13(9):2029. doi: 10.3390/microorganisms13092029 (PMC12472130; doi:10.3390/microorganisms13092029)
Supplement: Supplementary file 1 [file microorganisms-13-02029-s001.zip › Supplementary File S1. Supplementary Methods and Results.pdf]

# Viral quasispecies inference from single observations

Supplementary methods and results

Josep Gregori Miquel Salicrú Marta Ibáñez-Lligoña Sergi Colomer Castell
Carolina Campos Álvaro González-Camuesco Josep Quer

2025-06-18

## Abstract

RNA virus populations exist as quasispecies-complex, dynamic clouds of closely related but genetically diverse variants generated by high mutation rates during replication. Assessing quasispecies structure and diversity is crucial for understanding viral evolution, adaptation, and response to antiviral treatments. However, comparing single quasispecies observations from individual biosamples, especially at different infection or treatment time points, presents statistical challenges. Traditional inferential tests are inapplicable due to the lack of replicate observations, and resampling-based approaches such as the bootstrap and jackknife are limited by biases and non-independence, particularly for diversity indices sensitive to rare haplotypes. In this study, we address these limitations by applying the delta method to derive analytical variances for a set of quasispecies structure indicators specifically designed to assess quasispecies maturation. We demonstrate the utility of this approach using high-depth next-generation sequencing data from hepatitis C virus (HCV) quasispecies evolving in vitro under various conditions, including free evolution and exposure to antiviral or mutagenic treatments. Our results reveal that with highly fit HCV quasspecies, sofosbuvir inhibits quasispecies genetic diversity, while mutagenic treatments accelerate maturation, compared to untreated controls. We emphasize the interpretation of results through absolute differences, log-fold changes, and standardized effect sizes, moving beyond mere statistical significance. This framework enables robust, quantitative comparisons of quasispecies diversity from single observations, providing valuable insights into viral adaptation and treatment response.

## 1 Supplementary methods

### 1.1 Indicators' variance and standard error by the delta method

**Variance of the estimate of a proportion** from the binomial distribution:

$$\mathrm{SE}(\hat{p}_i)^2 = \frac{1}{n} \cdot \mathrm{Var}(\hat{p}_i) \approx \frac{1}{n} \cdot \hat{p}_i \cdot (1 - \hat{p}_i) \tag{1}$$

where $\hat{p}_i$ may be the master frequency, the aggregated frequencies of the top $N$ haplotypes, or that of all haplotypes below a given frequency, and $n$ is the sample size as reads number.

**Variance of the estimate of the ratio of two non-overlapping proportions**:

$$\text{SE}\left(\frac{\hat{p}_A}{\hat{p}_B}\right)^2 = \frac{1}{n}\text{Var}\left(\frac{\hat{p}_A}{\hat{p}_B}\right) \approx \frac{1}{n}\left[\frac{\hat{p}_A \cdot (1-\hat{p}_A)}{\hat{p}_B^2} + \frac{\hat{p}_A^2 \cdot (1-\hat{p}_B)}{\hat{p}_B^3} + \frac{2\;\hat{p_A}^2}{\hat{p}_B^2}\right] \tag{2}$$

**Variance of the estimate of top haplotype evenness as $R_k$**

With $R_k(\hat{P})$ defined as:

$$R_k(\hat{P}) = \frac{(k-1)\cdot\hat{p}_k}{\sum_{i=1}^{(k-1)}\hat{p}_i} = (k-1)\cdot\frac{\hat{A}}{\hat{B}} \tag{3}$$

where $\hat{A} = \hat{p}_k$ and $\hat{B} = \sum_{i=1}^{(k-1)}\hat{p}_i$, the variance is estimated as:

$$\text{SE}(R_k(\hat{P}))^2 = \frac{1}{n}\text{Var}(R_k(\hat{P})) \approx \frac{1}{n}(k-1)^2\left[\frac{\hat{A}(1-\hat{A})}{\hat{B}^2} + \frac{\hat{A}^2(1-\hat{B})}{\hat{B}^3} + 2\frac{\hat{A}^2}{\hat{B}^2}\right] \tag{4}$$

**Variance of the $\text{RLE}_\infty$ estimate**

Let us define $\text{RLE}_\infty(\hat{P})$ as:

$$\text{RLE}_\infty(\hat{P}) = \frac{\ln(1/\hat{p}_1)}{\ln(H)} = -\frac{\ln(\hat{p}_1)}{\ln(H)} \tag{5}$$

so that $\text{RLE}_\infty(\hat{P}) = 1$, when $\hat{p}_i = 1/H,\ \forall i = 1,\ldots,H$.

The standard error is estimated as:

$$\text{SE}(\text{RLE}_\infty(\hat{P}))^2 = \frac{1}{n}\text{Var}(\text{RLE}_\infty(\hat{P})) \approx \frac{1}{n}\left(\frac{1}{\ln(H)}\right)^2\left(\frac{1-\hat{p}_1}{\hat{p}_1}\right) \tag{6}$$

**Variance of the $\text{RLE}_1$ estimate**

Given:

$$\text{RLE}_1(\hat{P}) = \frac{\ln(D(\hat{P},1))}{\ln(H)} = \frac{\ln(\exp(\hat{H_S}))}{\ln(H)} = \frac{\hat{H_S}}{\ln(H)} \tag{7}$$

with $D(\hat{P},1)$ the Hill number of order $q=1$, $H$ number of haplotypes and $H_S$ Shannon entropy.

$$\text{SE}(\text{RLE}_1(\hat{P}))^2 = \frac{1}{n}\cdot\text{Var}(\text{RLE}_1(\hat{P})) \approx \frac{1}{n}\left(\frac{1}{\ln(H)}\right)^2\left[\sum_{i=1}^{H}\hat{p}_i(\ln(\hat{p}_i))^2 - \hat{H_S}^2\right] \tag{8}$$

**Variance of the $\text{RLE}_2$ estimate**

$$\text{RLE}_2(\hat{P}) = \frac{\ln(D(\hat{P},2))}{\ln(H)} = \frac{-\ln\left(\sum_{i=1}\hat{p_i}^2\right)}{\ln(H)} \tag{9}$$

$$\text{SE}(\text{RLE}_2(\hat{P}))^2 = \frac{1}{n}\text{Var}(\text{RLE}_2(\hat{P})) \approx \frac{1}{n}\left(\frac{2}{\ln(H)}\right)^2\left[\frac{\sum_{i=1}^{H}\hat{p_i}^3 - \left(\sum_{i=1}^{H}\hat{p_i}^2\right)^2}{\left(\sum_{i=1}^{H}\hat{p_i}^2\right)^2}\right] \tag{10}$$

**Variance of $d_A$ and $d_N$ estimates**

Given

$$d_A(f_1(P),\ldots,f_q(P)) = \sqrt{\sum_i^q [(f_i(P) - f_i(A)]^2} \tag{11}$$

the distance in the space of quasispeces indicators between a quasisecies sample with estimated vector of haplotype frequencies $P$ and the ideal quasispecies state A, where the $f_i$ are the set of quasispecies indicators, and $f_i(A)$ the indicator values of state A

The corresponding Taylor expansion of first order is:

$$d_A(f_1(\hat{P}),\ldots,f_q(\hat{P})) = d_A(f_1(P),\ldots,f_q(P)) + \sum_{i=1}^q \frac{\partial\, d_A(f_1(\hat{P}),\ldots,f_q(\hat{P}))}{\partial f_i(\hat{P})}\Bigg|_P (f_i(\hat{P}) - f_i(P)) + R_2$$

so that the standard error and the variance may be evaluated as

$$SE(d_A(f_1(\hat{P}),\ldots,f_q(\hat{P})))^2 = \frac{1}{n}\mathrm{Var}(d_A(f_1(\hat{P}),\ldots,f_q(\hat{P}))) \tag{12}$$

$$\mathrm{Var}(d_A(f_1(\hat{P}),\ldots,f_q(\hat{P}))) = \sum_{i,j=1}^q \frac{(f_i(\hat{P}) - f_i(A))(f_j(\hat{P}) - f_j(A))}{\sum_{k=1}^q (f_k(\hat{P}) - f_k(A))^2} \cdot \mathrm{Cov}(f_i(\hat{P}), f_j(\hat{(P)})) \tag{13}$$

or, expressed in matrix algebra:

$$\mathrm{Var}(d_A(F(\hat{P}))) = \frac{1}{\left\|F(\hat{P}) - F(A)\right\|^2} \times \left[(F(\hat{P}) - F(A))^T\ \mathrm{Cov}(F(\hat{P}))\ (F(\hat{P}) - F(A))\right] \tag{14}$$

where:

- $F(\hat{P}) = (f_i(\hat{P}), i = 1,\ldots,q)$ the vector of indicators estimates for the quasispecies with haplotype frequencies $P$,
- $F(A) = (f_i(A), i = 1,\ldots,q)$ the vector of indicator values for state A,
- $\mathrm{Cov}(F(\hat{P})) = (\mathrm{Cov}(f_i(\hat{P}), f_j(\hat{P})))$ is the $q \times q$ covariance matrix of the indicator functions,
- $\left\|F(\hat{P}) - F(A)\right\|^2 = (F(\hat{P}) - F(A))^T(F(\hat{P}) - F(A)) = \sum_{i=1}^q (f_i(\hat{P}) - f_i(A))^2$, the squared Euclidean norm of the difference vector.

and where

$$\mathrm{Cov}(f_i(\hat{P}), f_j(\hat{P})) = \mathrm{r}(f_i, f_j)\ \sqrt{\mathrm{Var}(f_i(\hat{P}))\mathrm{Var}(f_j(\hat{P}))}$$

or in matrix algebra notation:

Table S1: Cohen's $d$ interpretation guidelines

| $\mid d \mid$ | Magnitude | NOV$^{(1)}$ | PS$^{(2)}$ |
|---|---|---|---|
| (0.0 - 0.1] | Tiny | (0.000 - 0.040] | (0.500 - 0.528] |
| (0.1 - 0.2] | Very small | (0.040 - 0.080] | (0.528 - 0.556] |
| (0.2 - 0.5] | Small | (0.080 - 0.197] | (0.556 - 0.638] |
| (0.5 - 0.8] | Moderate | (0.197 - 0.311] | (0.638 - 0.714] |
| (0.8 - 1.2] | Large | (0.311 - 0.451] | (0.714 - 0.802] |
| (1.2 - 2.0] | Very large | (0.451 - 0.683] | (0.802 - 0.921] |
| $> 2.0$ | Huge | $> 0.683$ | $> 0.921$ |

(1)NOV: Non-overlap between the two distributions.
(2)PS: Probability of superiority. Probability that a randomly sampled individual from one group will have a higher score than a randomly sampled individual from the other group. It is a metric similar to the AUC.

$$\mathrm{Cov}(F(\hat{P})) = \left(\mathrm{diag}\left(\sqrt{Var(F(\hat{P}))}\right)\right) R(F) \ \left(\mathrm{diag}\left(\sqrt{Var(F(\hat{P}))}\right)\right) \tag{15}$$

with $\mathrm{R}(F) = (\mathrm{r}(f_i, f_j))$ the $q \times q$ intrinsic correlation matrix between indicator functions estimated empirically from a large dataset, and $\mathrm{diag}\left(\sqrt{Var(F(\hat{P}))}\right)$ the diagonal matrix with standard deviations of $F(\hat{P})$.

The variance of $d_N$ may be estimated as:

$$Var(d_N(f_1(\hat{P}), \ldots, f_q(\hat{P}))) = \left(\frac{1}{d_A(Z)}\right)^2 Var(d_A(f_1(\hat{P}), \ldots, f_q(\hat{P}))) \tag{16}$$

## 1.2 Effect size

Guidelines for interpreting Cohen's effect sizes, $d$, values using common terms of magnitude [1], and their probabilistic equivalents, non-overlap of distributions (NOV) and probability of superiority (PS) are given in Table S1, with:

$$\mathrm{NOV} = \left[1 - 2\Phi\left(\frac{-\mid d \mid}{2}\right)\right] \tag{17}$$

$$\mathrm{PS} = \Phi\left(\frac{\mid d \mid}{\sqrt{2}}\right) \tag{18}$$

where $\Phi$ represents the cumulative distribution function of the standard normal distribution. Note that in taking the absolute value of $d$ we ensure a minimum PS of 50%. The sign of $d$ will dictate which is the condition of superiority.

The two metrics may be approximately related using Taylor series expansion of first order as:

$$\mathrm{PS} \approx 0.5 + \frac{1}{\sqrt{2}}\,\mathrm{NOV}$$

### 1.3 RNA extraction, cDNA amplification and deep sequencing.

Total intracellular viral RNA was extracted from infected cells using the Qiagen RNeasy kit (Qiagen, Valencia, CA, USA), according to the manufacturer's instructions. RT-PCR was carried out using AccuScript (Agilent Technologies), with specific oligonucleotide primers. Negative controls without template RNA were included in parallel to ascertain the absence of cross-contamination by template nucleic acids. PCR products were purified (QIAquick Gel Extraction kit), quantified (Pico Green assay), and analyzed for quality (Bioanalyzer) prior to Illumina MiSeq sequencing.

### 1.4 NGS data treatment

The fastq files from MiSeq Illumina were treated to preserve full read integrity, completely covering the amplicon, to obtain amplicon-haplotypes and corresponding frequencies. Briefly, as previously described [2], full amplicon reads were obtained from the 2×300 bp paired-end reads with the help of FLASH [3], requiring a minimum overlap of 20 bp and a maximum of 10% mismatches; the reads accumulating more than 5% bp with Phred scores below Q30 were removed. The clean amplicon was finally obtained by trimming primers. Reads were collapsed into haplotypes and counts. For each amplicon a fasta file for each strand was obtained.

## 2 Supplementary results

### 2.1 Indicator's variances

Table S2: (A) - Variance of rarefied maturity indicators.

| ID | Var.Master | Var.Top25 | Var.Rare1 | Var.Rare2 | Var.dN |
|---|---|---|---|---|---|
| p0 | 0.19370 | 0.15409 | 0.17834 | 0.15428 | 0.09801 |
| p100 | 0.24997 | 0.18888 | 0.22045 | 0.17489 | 0.12216 |
| Ctl | 0.23907 | 0.18295 | 0.20046 | 0.18049 | 0.10993 |
| SOF | 0.23650 | 0.15225 | 0.17344 | 0.15618 | 0.10829 |
| RBV | 0.15037 | 0.24729 | 0.24977 | 0.23721 | 0.15826 |
| FPV | 0.10977 | 0.24344 | 0.24980 | 0.23383 | 0.28173 |

Table S2: (B) - Variance of rarefied maturity indicators.

| ID | Var.R5 | Var.R10 | Var.RLE1 | Var.RLE2 | Var.RLEinf | Var.dN |
|---|---|---|---|---|---|---|
| p0 | 0.10031 | 0.25204 | 0.17244 | 0.01958 | 0.00494 | 0.09801 |
| p100 | 0.30241 | 0.77045 | 0.16410 | 0.03843 | 0.01268 | 0.12216 |
| Ctl | 0.49267 | 0.35682 | 0.15524 | 0.03800 | 0.01891 | 0.10993 |
| SOF | 0.41794 | 0.27383 | 0.16279 | 0.02797 | 0.00825 | 0.10829 |
| RBV | 1.59694 | 2.08769 | 0.13902 | 0.06227 | 0.04624 | 0.15826 |
| FPV | 4.72389 | 2.82377 | 0.13175 | 0.05216 | 0.07329 | 0.28173 |

### 2.2 Tests

The changes in quasispecies structure indicators and in quasispeces maturity score are evaluated and tested between the following states:

- Pass 0 (p0) quasspecies versus pass 100 (p100), with no treatment in between (Table S3).
- Pass 110 (p110, Ctl), with extra 10 passes to p100, versus p100, with p100 used as treatment baseline (Table S4).

Table S3: Test p100 quasispecies versus p0. Dif: p100-p0, Ratio: p100/p0, adj.pval: BH adjusted p-values, d: Effect size, NOV: Non-overlap, PS: Probability of superiority.

| Feat | p0 | p100 | Dif | Ratio | t | adj.pval | d | NOV | PS |
|---|---|---|---|---|---|---|---|---|---|
| Master | 0.7373 | 0.4949 | -0.2424 | 0.6713 | -120.67 | 0.00e+00 | -0.5146 | 20.30 | 64.20 |
| Top25 | 0.8097 | 0.7472 | -0.0625 | 0.9228 | -35.38 | 0.00e+00 | -0.1508 | 6.01 | 54.25 |
| Rare1 | 0.2323 | 0.3281 | 0.0958 | 1.4124 | 50.31 | 0.00e+00 | 0.2145 | 8.54 | 56.03 |
| Rare2 | 0.1906 | 0.2259 | 0.0353 | 1.1853 | 20.42 | 0.00e+00 | 0.0871 | 3.47 | 52.45 |
| R5 | 0.0193 | 0.0502 | 0.0308 | 2.5938 | 16.11 | 0.00e+00 | 0.0687 | 2.74 | 51.94 |
| R10 | 0.0221 | 0.0598 | 0.0377 | 2.7088 | 12.37 | 0.00e+00 | 0.0527 | 2.10 | 51.49 |
| RLE1 | 0.2713 | 0.3907 | 0.1194 | 1.4401 | 68.26 | 0.00e+00 | 0.2910 | 11.57 | 58.15 |
| RLE2 | 0.0716 | 0.1485 | 0.0768 | 2.0724 | 105.78 | 0.00e+00 | 0.4511 | 17.84 | 62.51 |
| RLEinf | 0.0359 | 0.0784 | 0.0425 | 2.1847 | 106.23 | 0.00e+00 | 0.4530 | 17.92 | 62.56 |
| dN | 0.1753 | 0.2717 | 0.0965 | 1.5506 | 68.20 | 0.00e+00 | 0.2908 | 11.56 | 58.15 |

Table S4: Test p110 (Ctl) quasispecies versus p100. Dif: p110-p100, Ratio: p110/p100

| Feat | p100 | Ctl | Dif | Ratio | t | adj.pval | d | NOV | PS |
|---|---|---|---|---|---|---|---|---|---|
| Master | 0.4949 | 0.3954 | -0.0995 | 0.7990 | -47.18 | 0.00e+00 | -0.2012 | 8.01 | 55.66 |
| Top25 | 0.7472 | 0.7589 | 0.0117 | 1.0157 | 6.37 | 2.09e-10 | 0.0272 | 1.08 | 50.77 |
| Rare1 | 0.3281 | 0.2774 | -0.0507 | 0.8455 | -25.91 | 0.00e+00 | -0.1105 | 4.40 | 53.11 |
| Rare2 | 0.2259 | 0.2364 | 0.0104 | 1.0461 | 5.79 | 6.89e-09 | 0.0247 | 0.99 | 50.70 |
| R5 | 0.0502 | 0.0841 | 0.0339 | 1.6758 | 12.61 | 0.00e+00 | 0.0538 | 2.14 | 51.52 |
| R10 | 0.0598 | 0.0290 | -0.0307 | 0.4860 | -9.60 | 0.00e+00 | -0.0409 | 1.63 | 51.15 |
| RLE1 | 0.3907 | 0.4035 | 0.0128 | 1.0328 | 7.53 | 6.49e-14 | 0.0321 | 1.28 | 50.91 |
| RLE2 | 0.1485 | 0.1813 | 0.0328 | 1.2212 | 39.39 | 0.00e+00 | 0.1680 | 6.69 | 54.73 |
| RLEinf | 0.0784 | 0.1032 | 0.0248 | 1.3160 | 46.23 | 0.00e+00 | 0.1971 | 7.85 | 55.54 |
| dN | 0.2717 | 0.2926 | 0.0209 | 1.0768 | 14.37 | 0.00e+00 | 0.0613 | 2.44 | 51.73 |

- Ten passes of treatment with sofosbuvir (SOF), starting at p100, versus the base line p100 (Table S5).
- Ten passes of treatment with ribavrine (RBV), starting at p100, versus the base line p100 (Table S6).
- Ten passes of treatment with favipiravir (FPV), starting at p100, versus the base line p100 (Table S7).

Sofosbuvir is an inhibitor, a direct acting antiviral (DAA), Ribavirine and Favipiravir act as mutagens, increasing the virus replication error rate.

Table S5: Test the effect of 10 passes treated with Sofosbuvir, starting at p100. Dif: SOF-p100, Ratio: SOF/p100.

| Feat | p100 | SOF | Dif | Ratio | t | adj.pval | d | NOV | PS |
|---|---|---|---|---|---|---|---|---|---|
| Master | 0.4949 | 0.6162 | 0.1213 | 1.2450 | 57.67 | 0.00e+00 | 0.2459 | 9.78 | 56.90 |
| Top25 | 0.7472 | 0.8127 | 0.0654 | 1.0876 | 37.16 | 0.00e+00 | 0.1584 | 6.31 | 54.46 |
| Rare1 | 0.3281 | 0.2233 | -0.1048 | 0.6806 | -55.38 | 0.00e+00 | -0.2361 | 9.40 | 56.63 |
| Rare2 | 0.2259 | 0.1937 | -0.0322 | 0.8573 | -18.58 | 0.00e+00 | -0.0792 | 3.16 | 52.23 |
| R5 | 0.0502 | 0.0781 | 0.0279 | 1.5565 | 10.91 | 0.00e+00 | 0.0465 | 1.86 | 51.31 |
| R10 | 0.0598 | 0.0241 | -0.0357 | 0.4024 | -11.59 | 0.00e+00 | -0.0494 | 1.97 | 51.39 |
| RLE1 | 0.3907 | 0.3137 | -0.0770 | 0.8029 | -44.67 | 0.00e+00 | -0.1905 | 7.59 | 55.36 |
| RLE2 | 0.1485 | 0.1077 | -0.0408 | 0.7253 | -52.49 | 0.00e+00 | -0.2238 | 8.91 | 56.29 |
| RLEinf | 0.0784 | 0.0557 | -0.0227 | 0.7108 | -51.98 | 0.00e+00 | -0.2217 | 8.82 | 56.23 |
| dN | 0.2717 | 0.2081 | -0.0637 | 0.7656 | -44.01 | 0.00e+00 | -0.1876 | 7.47 | 55.28 |

Table S6: Test the effect of 10 passes treated with Ribavirine, starting at p100. Dif: RBV-p100, Ratio: RBV/p100.

| Feat | p100 | RBV | Dif | Ratio | t | adj.pval | d | NOV | PS |
|---|---|---|---|---|---|---|---|---|---|
| Master | 0.4949 | 0.1844 | -0.3106 | 0.3725 | -162.79 | 0.00e+00 | -0.6941 | 27.15 | 68.82 |
| Top25 | 0.7472 | 0.5521 | -0.1951 | 0.7389 | -97.99 | 0.00e+00 | -0.4178 | 16.55 | 61.62 |
| Rare1 | 0.3281 | 0.5152 | 0.1870 | 1.5701 | 90.47 | 0.00e+00 | 0.3858 | 15.29 | 60.75 |
| Rare2 | 0.2259 | 0.3869 | 0.1610 | 1.7125 | 83.17 | 0.00e+00 | 0.3546 | 14.07 | 59.90 |
| R5 | 0.0502 | 0.1689 | 0.1188 | 3.3677 | 28.58 | 0.00e+00 | 0.1219 | 4.86 | 53.43 |
| R10 | 0.0598 | 0.1140 | 0.0542 | 1.9075 | 10.64 | 0.00e+00 | 0.0454 | 1.81 | 51.28 |
| RLE1 | 0.3907 | 0.5792 | 0.1885 | 1.4825 | 113.56 | 0.00e+00 | 0.4842 | 19.13 | 63.40 |
| RLE2 | 0.1485 | 0.2906 | 0.1422 | 1.9576 | 148.58 | 0.00e+00 | 0.6336 | 24.86 | 67.29 |
| RLEinf | 0.0784 | 0.1729 | 0.0945 | 2.2049 | 129.07 | 0.00e+00 | 0.5504 | 21.68 | 65.14 |
| dN | 0.2717 | 0.4438 | 0.1720 | 1.6330 | 107.73 | 0.00e+00 | 0.4594 | 18.17 | 62.73 |

Table S7: Test the effect of 10 passes treated with Favipiravir, starting at p100. Dif: FPV-p100, Ratio: FPV/p100.

| Feat | p100 | FPV | Dif | Ratio | t | adj.pval | d | NOV | PS |
|---|---|---|---|---|---|---|---|---|---|
| Master | 0.4949 | 0.1255 | -0.3694 | 0.2536 | -204.26 | 0.00e+00 | -0.8710 | 33.68 | 73.10 |
| Top25 | 0.7472 | 0.5810 | -0.1662 | 0.7775 | -83.85 | 0.00e+00 | -0.3575 | 14.19 | 59.98 |
| Rare1 | 0.3281 | 0.5140 | 0.1859 | 1.5667 | 89.92 | 0.00e+00 | 0.3834 | 15.20 | 60.69 |
| Rare2 | 0.2259 | 0.3728 | 0.1469 | 1.6502 | 76.21 | 0.00e+00 | 0.3250 | 12.91 | 59.09 |
| R5 | 0.0502 | 0.4287 | 0.3785 | 8.5454 | 55.99 | 0.00e+00 | 0.2388 | 9.50 | 56.70 |
| R10 | 0.0598 | 0.1557 | 0.0959 | 2.6052 | 16.78 | 0.00e+00 | 0.0716 | 2.85 | 52.02 |
| RLE1 | 0.3907 | 0.5811 | 0.1904 | 1.4874 | 116.11 | 0.00e+00 | 0.4951 | 19.55 | 63.69 |
| RLE2 | 0.1485 | 0.3173 | 0.1688 | 2.1373 | 186.04 | 0.00e+00 | 0.7933 | 30.84 | 71.26 |
| RLEinf | 0.0784 | 0.2129 | 0.1345 | 2.7150 | 152.09 | 0.00e+00 | 0.6485 | 25.43 | 67.67 |
| dN | 0.2717 | 0.4755 | 0.2038 | 1.7499 | 106.35 | 0.00e+00 | 0.4535 | 17.94 | 62.58 |

Table S8: Summary of treatment results on dN, with dN.d.R the ratio of dN d values to the p100.vs.p0 dN d value

| Test | d | dN.d.R | dN.dif | dN.ratio | t | adj.pval | NOV | PS |
|---|---|---|---|---|---|---|---|---|
| p100.vs.p0 | 0.2908 | 1.0006 | 0.0965 | 1.5506 | 68.20 | 0.00e+00 | 11.56 | 58.15 |
| Ctl.vs.p100 | 0.0613 | 0.2109 | 0.0209 | 1.0768 | 14.37 | 0.00e+00 | 2.44 | 51.73 |
| SOF.vs.p100 | -0.1876 | -0.6456 | -0.0637 | 0.7656 | -44.01 | 0.00e+00 | 7.47 | 55.28 |
| RBV.vs.p100 | 0.4594 | 1.5806 | 0.1720 | 1.6330 | 107.73 | 0.00e+00 | 18.17 | 62.73 |
| FPV.vs.p100 | 0.4535 | 1.5603 | 0.2038 | 1.7499 | 106.35 | 0.00e+00 | 17.94 | 62.58 |

## 2.3 Comparing treatment effects on quasispecies indicators

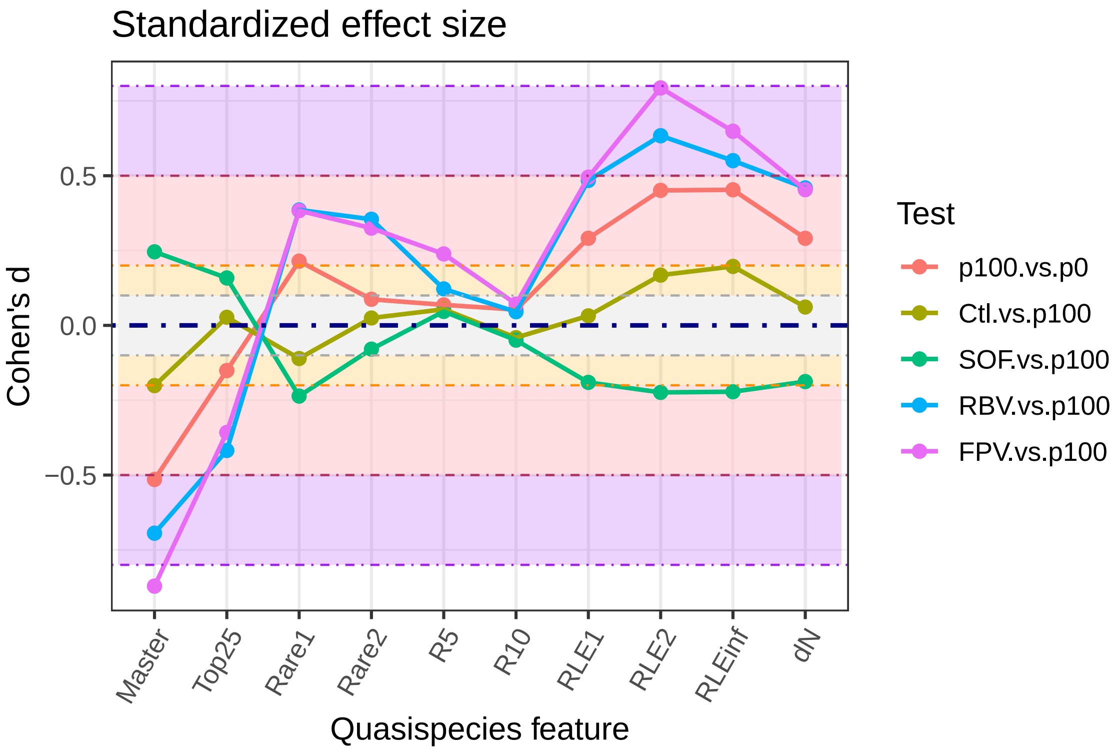


Figure S1: Cohens' d standardized effect size of treatments. Backgound color depicts effect magnitude regions. Gray: tiny effect, orange: very small effect, pink: small effect, purple: moderate effect, white: large effect.

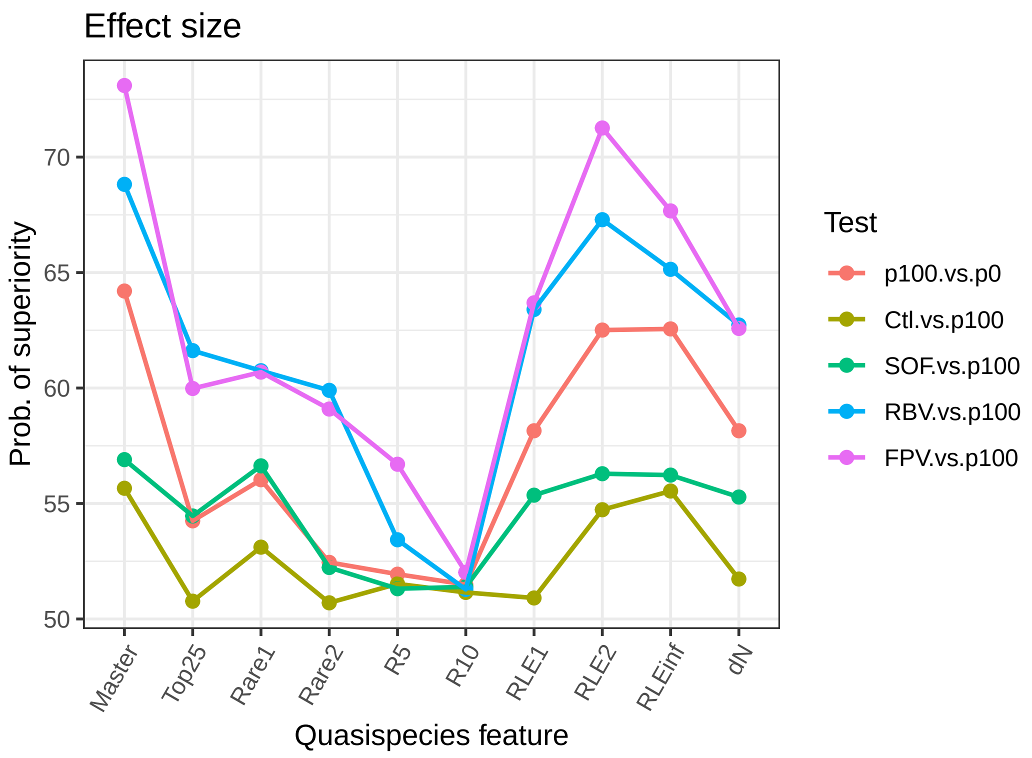


Figure S2: Standardized effect size of treatments, evaluated in probabilitistic terms as probability of superiority.

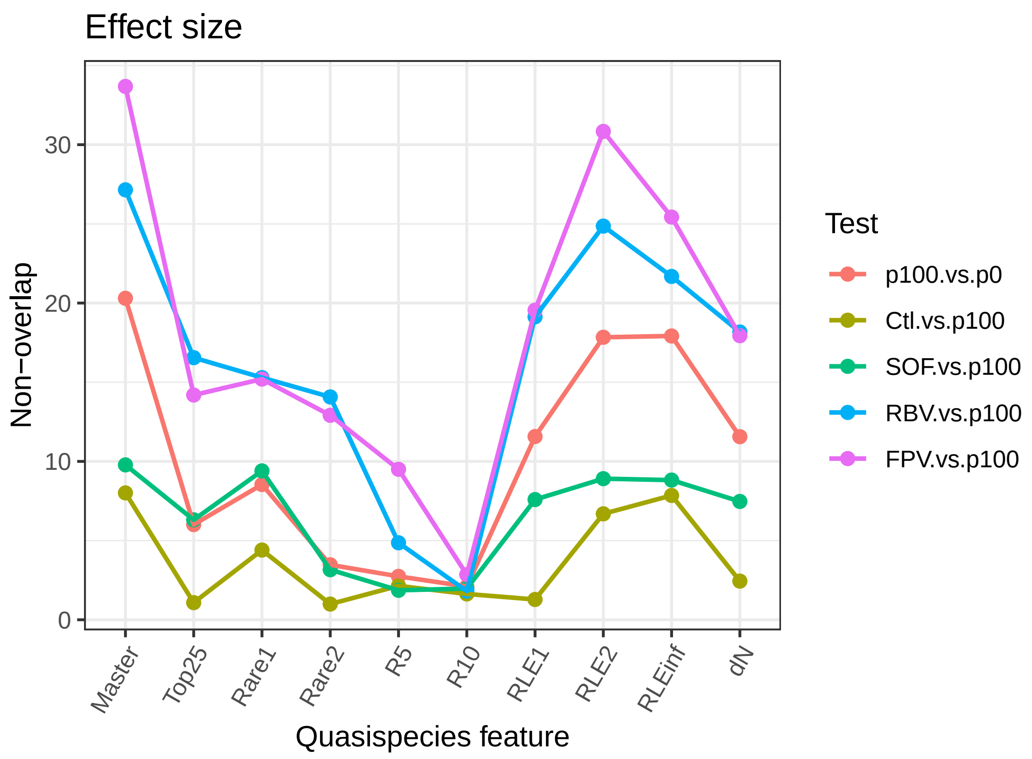


Figure S3: Standardized effect size of treatments, evaluated in probabilitistic terms as distributions non-overlap.

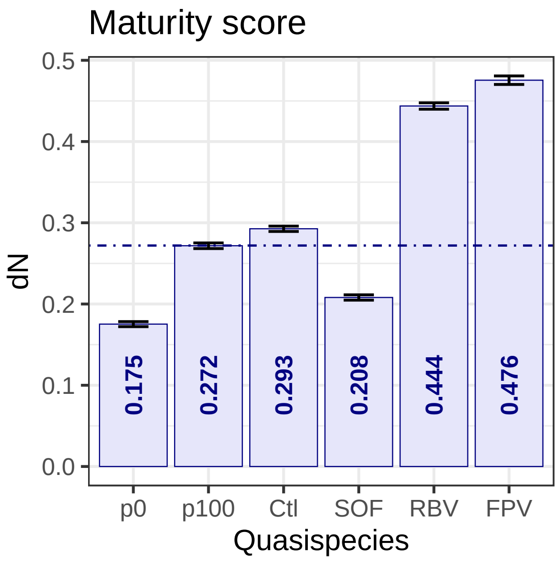


Figure S4: Maturity score as the normalized distance to state A. With 99.9% CIs. Dash-dot line at p100 dN as reference.

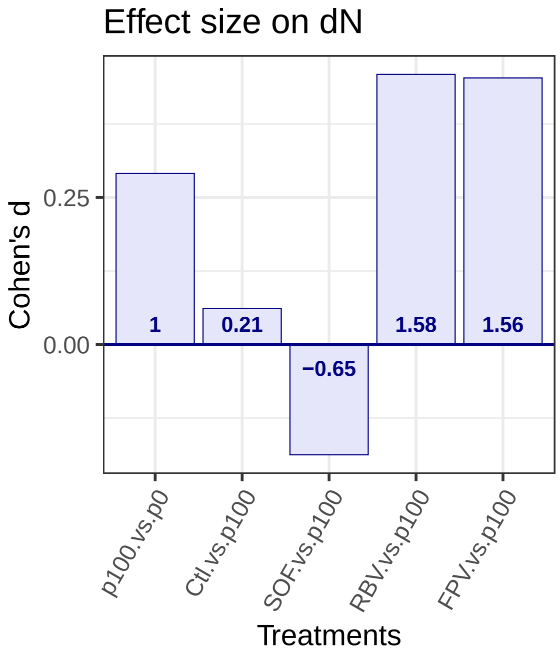


Figure S5: Effect size on dN in each treatment. Numbers inside bars: dN.d.R, ratio of dN d values to p100.vs.p0 d value.

# References


1. Sawilowsky, S. (2009). New effect size rules of thumb. Journal of Modern Applied Statistical Methods. 10.22237/jmasm/1257035100.

2. Colomer-Castell, S., Gregori, J., Garcia-Cehic, D., Riveiro-Barciela, M., Buti, M., Rando-Segura, A., Vico-Romero, J., Campos, C., Ibañez-Lligoña, M., Adombi, C.M., *et al.* (2023). In-host HEV quasispecies evolution shows the limits of mutagenic antiviral treatments. Int J Mol Sci *24*, 17185. 10.3390/ijms242417185.

3. Magoc, T., and Salzberg, S.L. (2011). FLASH: Fast length adjustment of short reads to improve genome assemblies. Bioinformatics *27*, 2957–2963. 10.1093/bioinformatics/btr507.

# Apendix A

## R code of main functions

```
### Get haplotype read counts from fasta file headers
#######################################################

get.nr <- function(fasta.flnm)
{
  seqs <- readDNAStringSet(fasta.flnm)
  parts <- t(sapply(names(seqs),
                  function(str) strsplit(str,split='\\|')[[1]]))
  rds <- as.integer(parts[,2])
  return(rds)
}

### Indicators' values and variances
###################################################################

###  Function computing selected diversity indices given the vector
###    of haplotype read counts of a quasispecies, nr
div.vals <- function(nr)
{
   p <- nr/sum(nr)
   p <- sort(p,decreasing=TRUE)

   H <- q_0 <- length(p)

   q_1 <- exp(-sum(p*log(p)))
   q_2 <- 1/sum(p^2)
   q_inf <- 1/p[1]

   RLE1 <- log10(q_1)/log10(q_0)
   Var.RLE1 <- (1/log(H))^2 * (sum(p*log(p)^2)-sum(p*log(p))^2)

   RLE2 <- log10(q_2)/log10(q_0)
   Var.RLE2 <- (2/log(H))^2 * (sum(p^3)-sum(p^2)^2)/(sum(p^2))^2

   RLEinf <- log10(q_inf)/log10(q_0)
   Var.RLEinf <- (1/log(H))^2 * (1-p[1])/p[1]

   Master <- p[1]
   Var.m <- p[1]*(1-p[1])

   Top25 <- sum(p[1:25])
   Var.top25 <- Top25*(1-Top25)

   Rare1 <- sum(p[p<0.01])
   Var.r1 <- Rare1*(1-Rare1)
   Rare2 <- sum(p[p<0.001])
   Var.r2 <- Rare2*(1-Rare2)
```

```
   k <- 5
   A <- p[k]
   B <- sum(p[1:(k-1)])
   R5 <- (k-1)*A/B
   Var.r5 <- (k-1)^2 * (A*(1-A)/B^2 + A^2*(1-B)/B^3 + 2*A^2/B^2)

   k <- 10
   A <- p[k]
   B <- sum(p[1:(k-1)])
   R10 <- (k-1)*A/B
   Var.r10 <- (k-1)^2 * (A*(1-A)/B^2 + A^2*(1-B)/B^3 + 2*A^2/B^2)


   return(c(q_0,Master,Top25,Rare1,Rare2,R5,R10,RLE1,RLE2,RLEinf,
            Var.m,Var.top25,Var.r1,Var.r2,Var.r5,Var.r10,Var.RLE1,Var.RLE2,
            Var.RLEinf))
}

all.cnms <- c('nHpl','Master','Top25','Rare1','Rare2',
      'R5','R10','RLE1','RLE2','RLEinf','Var.m','Var.top25',
   'Var.r1','Var.r2','Var.r5','Var.r10','Var.RLE1','Var.RLE2',
   'Var.RLEinf')

cnms <- c('nHpl','Master','Top25','Rare1','Rare2',
            'R5','R10','RLE1','RLE2','RLEinf')

###  States A and Z
A <- c(rep(1,2),rep(0,7))
names(A) <- cnms[-1]
Z <- c(rep(0,2),rep(1,7))
names(Z) <- cnms[-1]

### Distance A to Z
dAZ <- sqrt(sum((Z-A)^2))

### Distance from A
d2A.fn <- function(x)
{ x <- x[,names(A)]
  sqrt(sum((x[1,]-A)^2))
}

###  Rarefaction
################################################################

library(dqrng)

B <- 500  # Resampling cycles

###  B submostrejos aleatoris a 'size' reads, sense reposició,
###     i amb càlcul de diversitat
```

```
###
###      nr: vector amb nombre de reads per haplotip
###    size: mida de la submostra
###       B: nombre de rèpliques
### fn.div: funció de càlcul de diversitat a partir del vector de
###         reads per haplotip d'una submostra
###
###  B subsamples of 'size' reads, without replacement
###    with divrsity computation each time
###  nr: vector of reads per haplotype
subsample.no.rep <- function(nr,size,B,div.fn)
{ # Mapping of reads to haplotypes
  iHpl <- rep(1:length(nr),times=nr)
  # Sample size
  tnr <- sum(nr)

  # Get subsample and compute diversities
  one_time <- function()
  { # subsample reads
    s <- dqsample.int(tnr,size,replace=FALSE)
    # get haplotype indices
    vh <- iHpl[s]
    # vector of haplotype counts
    bs.rds <- as.integer(table(vh))
    # Compute diversity
    return(div.fn(bs.rds))
  }
  # Replicate B times
  subs.vals <- replicate(B, one_time(), simplify = "array")
  return(subs.vals)
}


### dA and dN variances from estimated indicators' correlation values
######################################################################

raref.d2A <- sapply(1:nrow(raref.vals),
                     function(i) d2A.fn(raref.vals[i,]))
raref.dN <- raref.d2A/dAZ

### Load correlation matrix
load("FullCohort-R.Var.Cov.RData")
Rf <- ind.R
ft <- setdiff(colnames(raref.vals),c('ID','nHpl','dN'))
Rf <- Rf[ft,ft]

ft.var <- setdiff(colnames(var.raref.vals),'ID')
Var.dA <- numeric(nrow(raref.vals))
names(Var.dA) <- raref.vals$ID
for(i in 1:nrow(raref.vals))
{
```

```
  est.Qs <- unlist(raref.vals[i,ft])
  v <- est.Qs-A
  norm.v <- t(v) %*% v

  var.Qs <- unlist(var.raref.vals[i,ft.var])
  Cov.Qs <- t(diag(sqrt(var.Qs))) %*% Rf %*% diag(sqrt(var.Qs))

  Var.dA[i] <- (1/norm.v) * (t(v) %*% Cov.Qs %*% v)
}
Var.dN <- (1/dAZ)^2 * Var.dA

raref.vals <- raref.vals %>%
     mutate(ID=factor(ID,levels=id.lvs)) %>%
  mutate(dN=raref.dN) %>%
  arrange(ID)

var.raref.vals <- var.raref.vals %>%
     mutate(ID=factor(ID,levels=id.lvs)) %>%
  mutate(Var.dN=Var.dN) %>%
  arrange(ID)


### Test between two rows, iA and iB, of the rarefied indicators matrix
########################################################################

qs_test <- function(iA,A.ID,iB,B.ID,raref.vals,var.raref.vals,n)
{
  # Estimates and variances
  est.A <- unlist(raref.vals[iA,] %>%
                  select(-c(ID,nHpl)))
  var.A <- unlist(var.raref.vals[iA,] %>%
                  select(-ID))/n
  est.B <- unlist(raref.vals[iB,] %>%
                  select(-c(ID,nHpl)))
  var.B <- unlist(var.raref.vals[iB,] %>%
                  select(-ID))/n

  # Test statistics
  num <- est.A-est.B
  denom <- sqrt(var.A+var.B)
  t <- num/denom
  d <- t * sqrt(2/n)

  vals <- raref.vals[c(iB,iA),] %>%
    select(-c(ID,nHpl)) %>% t()
    colnames(vals) <- c('B','A')

  vals <- vals %>% data.frame() %>%
    rownames_to_column(var='Feat') %>%
    relocate(Feat) %>%
    mutate( Dif=A-B,    # Observed difference
```

```
           Ratio=A/B,  # Observed ratio
           t=t,        # t or Z statistic
                       # Multi-test adjunted p-value
           adj.pval= p.adjust(2*(1-pnorm(abs(t))), method="BH"),
           d=d,        # Cohen's d
                       # Non-overlap
           NOV=round((1-2*pnorm(-abs(d)/2))*100,2),
                       # Probaility of superiority
           PS=round(pnorm(abs(d)/sqrt(2))*100,2))  %>%
    # Formatting
    mutate(t=round(t,2),
           adj.pval=sprintf("%.2e",adj.pval)) %>%
    dplyr::rename(!!A.ID := A, !!B.ID := B)
  return(vals)
}
```

Session info with libraries and versions

```
## R version 4.3.3 (2024-02-29 ucrt)
## Platform: x86_64-w64-mingw32/x64 (64-bit)
## Running under: Windows 11 x64 (build 22631)
##
## Matrix products: default
##
##
## locale:
## [1] LC_COLLATE=Catalan_Spain.utf8  LC_CTYPE=Catalan_Spain.utf8
## [3] LC_MONETARY=Catalan_Spain.utf8 LC_NUMERIC=C
## [5] LC_TIME=Catalan_Spain.utf8
##
## time zone: Europe/Madrid
## tzcode source: internal
##
## attached base packages:
## [1] stats4    stats     graphics  grDevices utils     datasets  methods
## [8] base
##
## other attached packages:
##  [1] dqrng_0.4.1         kableExtra_1.4.0    gridExtra_2.3
##  [4] lubridate_1.9.4     forcats_1.0.0       stringr_1.5.1
##  [7] dplyr_1.1.4         purrr_1.0.2         readr_2.1.5
## [10] tidyr_1.3.1         tibble_3.2.1        ggplot2_3.5.1
## [13] tidyverse_2.0.0     Biostrings_2.70.3   GenomeInfoDb_1.38.8
## [16] XVector_0.42.0      IRanges_2.36.0      S4Vectors_0.40.2
## [19] BiocGenerics_0.48.1
##
## loaded via a namespace (and not attached):
##  [1] gtable_0.3.6            xfun_0.49               tzdb_0.4.0
##  [4] vctrs_0.6.5             tools_4.3.3             bitops_1.0-9
##  [7] generics_0.1.3          fansi_1.0.6             pkgconfig_2.0.3
## [10] lifecycle_1.0.4         GenomeInfoDbData_1.2.11 compiler_4.3.3
## [13] farver_2.1.2            munsell_0.5.1           tinytex_0.54
```

```
## [16] htmltools_0.5.8.1      RCurl_1.98-1.16         yaml_2.3.10
## [19] pillar_1.9.0           crayon_1.5.3            tidyselect_1.2.1
## [22] digest_0.6.37          stringi_1.8.4           bookdown_0.41
## [25] labeling_0.4.3         fastmap_1.2.0           grid_4.3.3
## [28] colorspace_2.1-1       cli_3.6.3               magrittr_2.0.3
## [31] utf8_1.2.4             withr_3.0.2             scales_1.3.0
## [34] timechange_0.3.0       rmarkdown_2.29          hms_1.1.3
## [37] evaluate_1.0.1         knitr_1.49              viridisLite_0.4.2
## [40] rlang_1.1.4            Rcpp_1.0.13-1           glue_1.8.0
## [43] xml2_1.3.6             svglite_2.1.3           rstudioapi_0.17.1
## [46] R6_2.5.1               systemfonts_1.1.0       zlibbioc_1.48.2
```
